# Editorial: Comprehensive management and risk assessment of breast cancer-related lymphedema: a multidisciplinary approach

**DOI:** 10.3389/fonc.2026.1879730

**Published:** 2026-06-04

**Authors:** Joseph M. Escandón, Chihiro Matsui, Pedro Ciudad, Oscar J. Manrique

**Affiliations:** 1 Department of Surgery, Wyckoff Heights Medical Center, New York, NY, United States; 2 Department of Plastic and Reconstructive Surgery, Juntendo University School of Medicine, Tokyo, Japan; 3 Department of Plastic, Reconstructive and Burn Surgery, Arzobispo Loayza National Hospital, Lima, Peru; 4 Plastic Surgery, Ciruesthetic Clinic, Reconstructive and Burn Surgery, Lima, Peru; 5 Plastic Surgery, Austin-Weston Center for Cosmetic Surgery, Reston, VA, United States

**Keywords:** breast cancer lymphedema, lymph nodes, lymphedema, lymphedema/surgery, treatment outcome

Lymphedema is a chronic, progressive condition caused by impaired lymphatic drainage, leading to protein-rich fluid accumulation in tissue (1). Secondary lymphedema is the most common form in the United States, and as breast cancer survival rates have improved, the prevalence of breast cancer-related lymphedema (BCRL) has grown substantially (2). Affected patients experience upper limb swelling, pain, restricted movement, recurrent infections, and in severe cases, elephantiasis, alongside considerable psychosocial consequences such as anxiety, depression, and diminished body image — all of which significantly impair quality of life (2, 3).

Contemporary axillary management strategies have contributed to a reduction in the incidence of BCRL. Sentinel lymph node biopsy (SLNB), which minimizes disruption of the axillary lymphatic network compared with axillary lymph node dissection, is associated with significantly lower rates of postoperative lymphedema while maintaining accurate oncologic staging (2, 3). Furthermore, in carefully selected patients with early-stage, clinically node-negative breast cancer, emerging evidence supports the omission of sentinel node procedures altogether, thereby further reducing surgical trauma to the lymphatic system and potentially lowering the risk of BCRL without compromising oncologic outcomes (2, 3).

Treatment options span conservative approaches like compression therapy and physiotherapy to surgical interventions including lymphovenous anastomosis (LVA), vascularized lymph node transfer (VLNT), and liposuction for advanced disease (3, 4). Preventive strategies, particularly the LYMPHA technique performed during axillary lymph node dissection, have gained growing recognition. Nevertheless, outcomes remain limited once symptoms are established, and progressive lymphatic deterioration continues to challenge clinicians. Given that breast cancer is the most prevalent malignancy among women worldwide, BCRL represents a substantial clinical and humanistic burden (5). A thorough understanding of its pathophysiology, clinical presentation, and treatment options is essential to advancing evidence-based care. The articles in this Research Topic reflect the breadth and evolving sophistication of contemporary BCRL research and management.

The Research Topic opens with a comprehensive review by Knopf et al., which serves as an authoritative foundation for the topic. Their synthesis of the existing literature on BCRL pathophysiology, incidence, diagnostic strategies, and management guidelines—encompassing conservative, surgical, and emerging approaches—provides the clinical and scientific scaffold upon which the more specialized contributions build. This review underscores the necessity of standardized, multidisciplinary care pathways, and informed patient counseling as essential pillars of BCRL management.

A recurring and clinically critical theme across this Research Topic is the imperative for early risk identification. Chen et al. address postoperative seroma, an underappreciated precursor to delayed lymphatic recovery, through the development and validation of a nomogram-based predictive model. Employing Cox proportional hazards regression in a dual-center retrospective cohort of 373 patients, they identified body mass index, concomitant diabetes mellitus, modified radical mastectomy, and extent of lymph node dissection as independent predictors of seroma resolution. The model demonstrated strong discriminatory performance, with area under the curve values reaching 0.90 in the independent validation cohort on postoperative day 7, and favorable calibration and clinical utility on decision curve analysis. This tool represents a practical instrument for guiding individualized postoperative care and early intervention in high-risk patients.

Complementing this work, Peng et al. present a machine learning-based personalized risk prediction model for BCRL itself, integrating multimodal clinical and behavioral data from 368 postoperative breast cancer patients. Following feature selection via LASSO regression, nine algorithms were benchmarked, with logistic regression emerging as optimal, achieving an AUC of 0.937 in the validation set. Shapley Additive Explanations (SHAP) enabled granular interpretation of model outputs, identifying BMI, lymph node dissection level, and nodal status as the dominant contributors. Together, these two modeling studies establish a compelling evidence base for algorithm-assisted, personalized risk stratification from the immediate postoperative period through long-term follow-up.

Turning to intervention, Sun et al. conducted a systematic review and meta-analysis of physical therapy modalities for BCRL prevention and treatment, synthesizing data from 31 studies encompassing 4,323 patients. While the pooled analyses did not demonstrate statistically significant preventive or therapeutic effects overall, subgroup analysis revealed a significant reduction in BCRL incidence with interventions sustained for 12 months (RR = 0.53, 95% CI 0.29–0.95), a finding with direct implications for clinical program design. This result highlights the critical importance of intervention duration and argues for sustained, longitudinal rehabilitation programs rather than short-term protocols.

Lommer et al. extend the therapeutic landscape by mapping the evidence for pharmacologic and herbal agents in BCRL prevention and treatment through a systematic scoping review of 24 studies. Their findings are notably cautious: NSAIDs, anti-hypertensives, and thiazolidinediones demonstrated no benefit, while data on flavonoid-derived venoactive agents and herbal compounds remained inconclusive. Nevertheless, two promising signals emerged: glucagon-like peptide-1 receptor agonists (GLP-1 RAs) were associated with reduced BCRL incidence, and immunomodulatory therapies showed benefit in symptom management. These observations, while preliminary, open new investigative avenues, particularly given the established systemic and anti-inflammatory profiles of GLP-1 RAs.

At the molecular level, Dong et al. provide a novel contribution through integrated RNA sequencing, machine learning, and clinical data analysis of adipose tissue from 40 patients with cancer-associated secondary lymphedema. Three genes—IL2RG, HOXD10, and TSPAN1—were identified as candidate biomarkers, with diagnostic models built on these markers demonstrating excellent performance. Immune infiltration analyses and single-cell transcriptomics further revealed the centrality of T cell and macrophage interactions in BCRL pathogenesis. The validation of IL2RG expression by RT-qPCR and its association with clinical parameters positions these findings as a meaningful step toward molecular diagnostics and targeted therapeutic development.

Finally, He et al. address the often-neglected psychosocial dimension of BCRL by investigating depression risk among 120 patients with postoperative lymphedema. With a depression incidence of 43.33%, their study underscores the profound psychological burden of this condition. Multivariate analysis identified low family income, poor body image, and diminished social support as independent risk factors. Although the external validation AUC of 0.629 reflects modest predictive accuracy, the clinical significance of these findings is clear: comprehensive BCRL management must incorporate systematic mental health screening and psychosocial support, particularly for socioeconomically vulnerable patients.

Collectively, these contributions delineate a research agenda that is simultaneously broad and urgent. From molecular biomarker identification and machine learning-based risk stratification, to the optimization of physical therapy duration and the exploration of novel pharmacological targets, this Research Topic advances the field across multiple dimensions. The consistent call for prospective, standardized, and longer-term studies reflects a shared recognition that the current evidence base, though growing, remains insufficient to fully guide clinical practice. It is our hope that this Research Topic will stimulate further interdisciplinary collaboration, inspire rigorous trial design, and ultimately translate into meaningfully improved outcomes for the millions of breast cancer survivors living with lymphedema worldwide.
